# Effect of Pupal Cold Storage on Reproductive Performance of *Microplitis manilae* (Hymenoptera: Braconidae), a Larval Parasitoid of *Spodoptera frugiperda* (Lepidoptera: Noctuidae)

**DOI:** 10.3390/insects13050449

**Published:** 2022-05-09

**Authors:** Binglin Xing, Lei Yang, Ahamaijiang Gulinuer, Fen Li, Shaoying Wu

**Affiliations:** 1 Sanya Nanfan Research Institute, Hainan University, Sanya 572024, China; bing425318472@163.com (B.X.); or yanglei@zju.edu.cn (L.Y.); huahuanur@163.com (A.G.); lifen2010happy@sina.com (F.L.); 2 College of Tropical Crops, Hainan University, Haikou 570228, China; 3 Institute of Insect Sciences, Zhejiang University, Hangzhou 310058, China

**Keywords:** *Spodoptera frugiperda*, parasitoid, *Microplitis manilae*, cold storage, biological control

## Abstract

**Simple Summary:**

Parasitoids are one of the most important biological control agents, and there are increasing requirements for long-term breeding. It is critical to figure out the parasitoid biological properties and disclose the effects of cold storage on them to extend their longevity. In this study, we investigated the field parasitism rate and clarified the biological parameters of *Microplitis manilae*, a dominant larval parasitoid of *Spodoptera frugiperda*. Further analysis revealed that the pupal cold storage, including different storage temperatures, storage period and storage time, significantly affected the emergence rate, parasitism rate and longevity of wasp adults, and the optimal storage condition was middle-aged pupae stored at 10 °C for 5–10 d. These results provide a novel insight into the mass-rearing of *M. manilae* and contribute to the biological control using *M. manilae* against *S. frugiperda*.

**Abstract:**

As a major invasive pest in China, *Spodoptera frugiperda* (Smith) (Lepidoptera: Noctuidae) has caused great damage to crops. Hymenopteran parasitoids, especially the braconid wasps, play crucial roles in depressing pest populations. However, there was little information about the ideal storage of parasitoids to achieve their mass-rearing. Here, we identified a dominant parasitoid of *S. frugiperda*, *Microplitis manilae* (Ashmead) (Hymenoptera: Braconidae), in the Hainan province of China with a field parasitism rate of 5.66–19.10%. The investigation of biological parameters revealed that the parasitism rate of *M. manilae* significantly decreased with an increase in both wasp adult longevity and host age, and the wasp of 1–3 d post eclosion performed best on the first instar of host larvae, showing the highest parasitism rate. We also discovered that the decreased temperature from 30 to 20 °C greatly extended the longevity of wasp adults, and a similar result was observed after feeding on 10% sucrose water compared with sterile water. Then, the effects of different pupal cold storage temperatures (4 and 10 °C), storage period (prepupa, middle-aged pupa, late-aged pupa) and storage time (5, 10 or 20 d) on the emergence rate, parasitism rate, female proportion and longevity of *M. manilae* were investigated. The results demonstrated that the middle-aged wasp pupae stored at 10 °C for 5–10 d possessed a stronger parasitic ability and longer longevity. These findings may promote the flexibility and efficacy of large-scale production of *M. manilae*, thus contributing to its biological field control against *S. frugiperda.*

## 1. Introduction

As a prominent invasive pest in tropical and subtropical regions, the fall armyworm (FAW), *Spodoptera frugiperda* (Smith) (Lepidoptera: Noctuidae) feeds on a broad variety of plants, including at least 353 species belonging to 76 families, such as maize, wheat, rice and sorghum, which result in huge economic losses to agriculture [1]. Since the beginning of 2019, FAW has rapidly colonized the cornfields of Yunnan Province in southern China, and its invasion in Hainan province was first detected on 30 April 2019. At present, *S. frugiperda* is still primarily controlled by chemical pesticides, albeit the field populations have developed high levels of resistance to various insecticides [2,3,4], of which the detoxification genes were mainly involved [5]. Biological control is one of the most essential methods in long-term pest management due to its great advantages, such as a persistent effect, low cost, without insecticide resistance and pesticide residues, thus offering considerable ecological, economic and social benefits [6].

*Microplitis manilae* (Ashmead) (Hymenoptera: Braconidae) is a solitary endoparasitoid parasitizing several lepidopteran pests, of which the species of *Spodoptera* are mainly preferred [7,8,9,10,11]. As an excellent biological control agent, *M. manilae* was first identified in the Philippine Islands [12]. In China, it was found that *M. manilae* showed great potential to parasitize the first to third instar larvae of both *Spodoptera exigua* (Hiibner) and *Spodoptera litura* (Fabricius) in the investigations of 2006 and 2010 [7,13]. Further, several lines of evidence suggested that the dominant parasitic species of both *S. exigua* and *S. litura* was *M. manilae* in Japan [7], Pakistan [8] and Vietnam [9]. Previous research has established that *M. manilae* had a distinct advantage in the interspecific competition after jointly releasing with the egg-larval parasitoid *Chelonus insularis* (Cresson) (Hymenoptera: Braconidae) [14]. Moreover, *M. manilae* parasitization inhibited the host feeding and subsequently led to the growth retardation of host insects [15]. To sum up, *M. manilae* is a promising biological agent for the control of noctuid pests.

The accumulation of sufficient storage methods is necessary for large-scale production and field release of natural enemies [16], and it is of extreme urgency to discover novel methods to extend the shelf life of wasps. Among which cold storage has been commonly used [17], providing us with an optional way to maintain a stable and sufficient wasp population. It has been well established that low temperatures retarded insect development [18], which allows us to synchronously release the wasps according to the pest occurrence regularity. Although there are existing advantages, keeping parasitoids at sub-ambient temperatures adversely affects the fitness of some parasitoids [19,20]. Therefore, to minimize the parasitoid reproductive performance loss after cold storage, it is necessary to investigate the effect of cold storage on the biological parameters of parasitoids, which contributes to establishing technical constraints for their commercial production and application [21,22,23].

There was an abundance of studies that have been implicated in the effects of cold storage on parasitoid biological parameters, including the species of Trcihogrammatidae and Braconidae, such as *Trichogramma evanescens* (Westwood) [24], *Trichogramma chilonis* (Ishii) [25], *Bracon hebetor* (Say) [26] and *Aphidius picipes* (Nees) [27]. Cold storage caused a declined survival rate, survival time and fecundity of wasp adults in *Gonatocerus ashmeadi* (Girault) [28,29], *Anaphes ovijentatus* (Crosby & Leonard) [30], *B. hebetor* [26] and Aphidiine parasitoids [31]. Moreover, low-temperature stress also affected the proportion of fertilized eggs [32], thus leading to the changes in sex ratio [33]. In parallel, numerous studies have demonstrated that host recognition, egg-laying amount and other parasitic behaviors of parasitoids altered after chilling treatment, such as in *A. picipes* [27] and *Anaphes victus* (Huber) [34]. Conversely, it has also been revealed that the emergence rate of wasp adults was unaffected after pupal cold storage in *B. hebetor* [26], *Aphidius matricariae* (Haliday) [35], *Lysiphlebus testaceipes* (Cresson) [36], *Encarsia formosa* (Gahan) [37] and *Muscidifurax raptor* (Girault & Sanders) [38]. Altogether, although much of the research up to date has been conducted, no researchers have been able to draw on any systematic research into the effect of cold storage on biological parameters of *M. manilae*, one of the prevalent parasitoids of noctuid pests.

In this study, we identified a dominant larval parasitoid of *S. frugiperda*, *M. manilae*, in the cornfield of Hainan Province of China in 2020 and investigated the field parasitism rate followed by the determinations of adult longevity and parasitism ability of *M. manilae*. Subsequently, we evaluated the effects of different pupal storage temperatures (4 and 10 °C), storage period (prepupa, middle-aged pupa, late-aged pupa) and storage time (5, 10 and 20 d) on the emergence rate, adult longevity and parasitism ability of *M. manilae*. We speculate that the reproductive performance of *M. manilae* can be improved with the increasing temperature and decreasing time under pupal cold storage, and the middle-aged pupal storage at 10 °C is possibly the most promising strategy for mass rearing. It is hoped that these findings will accelerate the large-scale production of *M. manilae*, thus contributing to its application in the biological control programs against *S. frugiperda*.

## 2. Materials and Methods

### 2.1. Insect Rearing

*M. manilae* and *S. frugiperda* were originally collected from the cornfield located in Yazhou District of Sanya city in Hainan province of China (109°11′ E, 18°22′ N) in 2020. We identified the *M. manilae* by morphological and molecular methods using their *COI* gene (Taxonomy ID: 1427173), which was the first discovery in the Hainan Province of China. A laboratory colony of *S. frugiperda* was established by feeding on an artificial diet made of corn meal, soybean meal and agar (Patent no. 201921652702.2) [39]. *S. frugiperda* were continuously raised in the laboratory for two years (at least 30 generations). Once eclosion, the adults were fed with 10% sucrose water (m/m) and allowed to lay eggs on a piece of gauze. The eggs were collected in a transparent box covered with 200 mesh nets, and the hatched larvae were reared until pupation. For *M. manilae*, we fed the adults with 10% sucrose water post eclosion followed by being fully mated for 24 h. *S. frugiperda* first instar larvae were used for 24 h parasitization to achieve a maximum indoor breeding efficiency with a 1:30 ratio (parasitoid: host). *M. manilae* was continuously bred in the laboratory for at least 20 generations. Both *M. manilae* and *S. frugiperda* were reared under controlled conditions of 25 ±1 °C, 65 ±5% relative humidity (RH) and 16:8 h (L: D). We used the laboratory colonies of *S. frugiperda* and *M. manilae* in subsequent analyses.

### 2.2. Investigation of Field Parasitism Rate

The larvae of *S. frugiperda* were respectively collected in five cities of Hainan Province, namely Qiongzhong with an average annual temperature of 23.2 °C, 79–88% RH and 2444 mm rainfall, Danzhou with an average annual temperature of 23.3 °C, 77–86% RH and 1815 mm rainfall, Sanya with an average annual temperature of 25.5 °C, 77–87% RH and 1279 mm rainfall, Ledong with an average annual temperature of 25.3 °C, 77–87% RH and 1600 mm rainfall and Dongfang with an average annual temperature of 24.5 °C, 72–81% RH and 1150 mm rainfall, during October and November of 2020, and in May 2021 (www.hainanqx.cn). The collected larvae were kept at 25 °C until the eclosion of either *S. frugiperda* or parasitoids to determine the field parasitism rate of *M. manilae* against *S. frugiperda*. The field parasitism rate (%) was calculated as (number of parasitized *S. frugiperda* larvae/total number of collected larvae) × 100.

### 2.3. Experimental Set-Up of M. manilae Pupal Cold Storage

The *M. manilae* within 12 h pupariation was maintained at 25 °C for 1, 3 and 5 d to obtain prepupa, middle-aged pupa and late-aged pupa, which were subsequently stored in an artificial climate chamber (YangHui, Ningbo, China) for 5, 10 and 20 d at 4 and 10 °C, respectively. Each treatment included at least 15 cocoons. The adults that emerged from the treated cocoons were considered F1, which were used to examine the effect of pupal cold storage on F1 emergence rate, survival time and parasitism rate, and 26 1st instar larvae of *S. frugiperda* (the maximum parasitized number) were used for 24 h parasitization per F1 female adult after being fully mated. The emerged wasp adults were regarded as F2 generations, followed by the determinations of emergence rate and female proportion in F2. Three repetitive experiments were conducted. *M. manilae* cocoons without pupal cold storage were reared under the optimal conditions of 25 ± 1 °C, 65 ± 5% RH and 16:8h (L: D), and the emerged parasitoids were set as the control.

### 2.4. Determinations of Parasitoid Emergence Rate and Female Proportion

After pupal cold storage, the number of emerged F1 adults was counted. We calculated the F1 emergence rate (%) as (number of emerged parasitoids/total number of wasp cocoons after pupal cold storage) × 100. The F1 female proportion (%) was calculated as (number of emerged female adults/total number of emerged parasitoids) × 100. Each treatment contains at least 15 cocoons of *M. manilae*. To determine the emergence rate and female proportion of F2, F1 females after being fully mated (*n* ≥ 15) were used for subsequent parasitization and these two parameters were calculated as above. Three repetitions were performed.

### 2.5. Determination of Parasitism Rate

To evaluate the parasitism ability of *M. manilae* at different developmental stages that were raised at optimal conditions without pupal cold storage, we used the female adults of 1, 3, 5, 7 and 9 d post-emergence after being fully mated and the host *S. frugiperda* of different ages, including 1st, 2nd, 3rd and 4th instar larvae, were collected for 24 h after being parasitized at 25 °C. For pupal cold storage treatment, the F1 wasp female adults post 1–3 d eclosion were used for parasitizing the 1st larvae of *S. frugiperda* for 24 h to evaluate the parasitism ability of F1 *M. manila* after pupal cold storage. The parasitism rate (%) was calculated as above. A total of 15–20 replicates were performed in this experiment, and each repetition contained 1 female adult of *M. manila* and 26 1st larvae of *S. frugiperda* (the maximum parasitized number).

### 2.6. Determination of Parasitoid Longevity

To clarify the effects of different temperatures and foods on the longevity of *M. manilae* adults raised at optimal conditions, at least 15 adults of *M. manilae* without pupal cold storage were collected and respectively raised at 20, 22.5, 25, 27.5 and 30 °C under conditions of 65 ± 5% RH and 16:8 h (L:D) after emergence. All adults were fed with 10% sucrose water or sterile water (control). The number of dead parasitoids was recorded every day. To evaluate the longevity of *M. manilae* F1 adults after pupal cold storage, at least 15 emerged F1 wasps were raised with 10% sucrose water under conditions of 25 °C, 65 ± 5% RH and 16:8 h (L:D). The number of dead parasitoids in F1 was recorded every day. We calculated the average longevity as (total survival time of wasp adults/total number of wasp adults). Three repetitions were performed in this experiment.

### 2.7. Statistical Analysis

All data were analyzed by SPSS software (SPSS, New York, NY, USA). Before analysis, the percentage data were arcsine square root-transformed to fit a normal distribution. The parasitism rate (response variable) was analyzed using one-way ANOVA in which the host instars and age of parasitoids were set as independent variables. The adult longevity, emergence rate and female proportion of F1 and F2 *M. manilae* adults (response variables) were analyzed using univariate two-way ANOVA (generalized linear model, GLM), in which the storage temperature and storage period of wasp cocoons were set as independent variables. When the means were not normally distributed, a nonparametric Kruskal–Wallis H test was used to determine the F1 parasitism rate after pupal cold storage (response variable) and independent variables were set as above. Statistical significances were marked with different letters (a, b, c, …). We plotted all figures using GraphPad Prism 7.0 software (San Diego, CA, USA).

## 3. Results

### 3.1. Field Parasitism Rate and Biological Parameters of M. manilae

#### 3.1.1. Field Parasitism Rate Investigation of *M. manilae*

In 2020–2021, the *S. frugiperda* larvae parasitized by *M. manilae* were collected, revealing varied field parasitism rates in different locations (Table 1). In detail, the highest parasitism rate was recorded in Dongfang city, reaching 19.10%, followed by Ledong1 with a 13.74% parasitism rate in 2020. In comparison, there was a lower field parasitism rate of 5.66% in Ledong2 in 2020. From the results in Table 1, we also observed that the field parasitism rate of *M. manilae* reached 8.09% in Danzhou in 2021. However, *M. manilae* failed to parasitize the *S. frugiperda* population of Sanya.

#### 3.1.2. Effects of Food and Temperature on Adult Longevity of *M. manilae*

**Figure 1:**
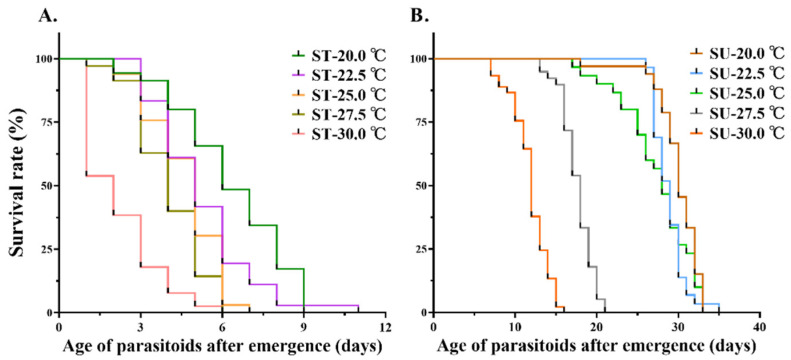
Effects of food on adult longevity of *M. manilae*. (**A**) Survival curves of *M. manilae* adults upon feeding on sterile water at different temperatures. (**B**) Survival curves of *M. manilae* adults upon feeding on 10% sucrose water at different temperatures. SU means 10% sucrose water, and ST represents sterile water (*n* ≥ 15).

Table 2 presented the longevity exposition of *M. manilae*, which revealed that feeding on 10% sucrose water significantly prolonged the wasp longevity from 6.31 to 29.66 d compared with that of sterile water at 20 °C. Closer inspection of the results showed that the longevity of *M. manilae* remarkably shortened with the increase of temperature from 20 to 30 °C, and there was a significant difference (F = 206.05; *df* = 6, 98; *p* < 0.001) between the treatment of 20 (29.66 d) and 30 °C (12.26 d). We also found that the survival rate of *M. manilae* decreased significantly as the temperature increased (Figure 1).

#### 3.1.3. Determination of Parasitism Rate of *M. manilae*

**Figure 2:**
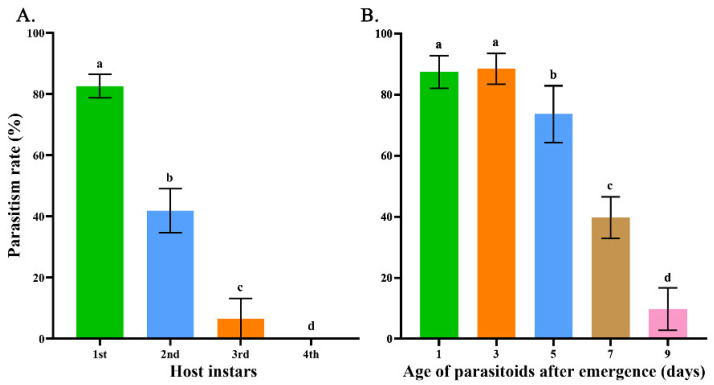
The parasitism rates of *M. manilae* on different instars of *S. frugiperda* larvae at different developmental stages. (**A**) The parasitism rates of *M. manilae* post 1–2 d eclosion on first, second, third and fourth *S. frugiperda* larvae (*n* ≥ 15). (**B**) The parasitism rates of *M. manilae* at 1, 3, 5, 7 and 9 d post-eclosion on *S. frugiperda* first larvae (*n* ≥ 15). One-way ANOVA between different groups was performed followed by Tukey’s multiple comparison tests, and the different letters mean significant differences (*p* < 0.05). Data are presented as mean ± SE.

The next investigation was concerned with the parasitic ability of *M. manilae* on different instars of host *S. frugiperda* larvae at different developmental stages. What stood out in Figure 2A was that the parasitism rate of *M. manilae* was significantly reduced with the increase in the host larval age. *M. manilae* had the greatest potential to parasitize *S. frugiperda* first larvae with a parasitism rate of 82.58% compared to those of 41.86% and 10.86% in second and third larvae, respectively (F = 1090.50; *df* = 3, 64; *p* < 0.001). Moreover, *M. manilae* failed to parasitize the fourth larvae of host *S. frugiperda*.

The following step was to determine the parasitic ability of *M. manilae* at different developmental periods on the first larvae of host *S. frugiperda*. The results, as shown in Figure 2B, indicate that the parasitoid adult after 1–3 d eclosion performed best on *S. frugiperda* larvae with a parasitism rate of 87.44–88.46%. With an increase in the wasp age, there was a significant decline in parasitism rate, merely reaching 9.74% in female wasp adults after 9 d eclosion (F = 374.82; *df* = 4, 80; *p* < 0.001).

### 3.2. Effects of Pupal Cold Storage on the Biological Parameters of M. manilae

#### 3.2.1. Determination of *M. manilae* Emergence Rate

From Table 3, we recorded that the emergence rate of *M. manilae* significantly differed upon different treatments of storage temperature, storage time and pupal age. Generally, the emergence rates of parasitoid adults significantly decreased with a temperature reduction from 10 to 4 °C and a prolonged time from 5 to 10–20 d after prepupa, middle-aged and late-aged pupal cold storages. Notably, there was a significantly higher emergence rate in wasp adults upon middle-aged pupal chilling stress compared to those of prepupa and late-aged pupal cold storage schemes at 4 and 10 °C (Table 3). However, the emergence rates of pupal cold storage treatments were significantly lower than that of the control group (F: 108.95–1027.39; *df* = 6, 15; *p* < 0.001). The most striking result was that 91.88% of wasps successfully emerged after the middle-aged pupal chilling at 10 °C for 5 d, presenting a minimum difference compared with the control group.

#### 3.2.2. Determination of *M. manilae* Parasitism Rate

Based on the above results, we recorded relatively low emergence rates after 20 d cold storage, which was excluded from the subsequent analysis. The *M. manilae* adults, after pupal treatments under a lower storage temperature or a prolonged storage time, showed a remarkable decreased parasitism rate on the first instar of host *S. frugiperda* (Table 4). Furthermore, the parasitism rates of wasp adults emerging from middle-aged pupal cold storage was significantly higher than those of prepupa (H = 83.12; *df* = 6; *p* < 0.001) and late-aged pupal treatments (H = 69.64; *df* = 6; *p* < 0.001) under the same storage schedules (Table 4). We further obtained a remarkable outcome that the *M. manilae* adults after middle-aged pupal cold storage at 10 °C for 5 d performed best on host *S. frugiperda*, showing a relatively high parasitism rate of 69.23%.

#### 3.2.3. Determination of *M. manilae* Adult Longevity

**Figure 3:**
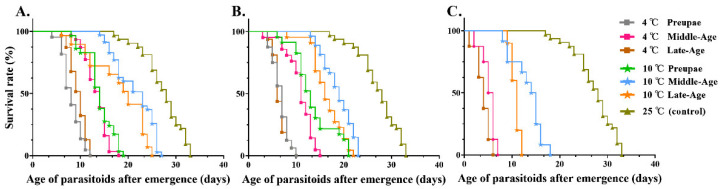
Survival curves of *M. manilae* adults upon different pupal cold storage treatments for different times. (**A**) Survival curves of *M. manilae* adults upon different pupal cold storage for 5 d (*n* ≥ 15). (**B**) Survival curves of *M. manilae* adults upon different pupal cold storage for 10 d (*n* ≥ 15). (**C**) Survival curves of *M. manilae* adults upon different pupal cold storage for 20 d (*n* ≥ 15).

The most striking result observed in Table 5 was that pupal cold storage had a significant effect on the longevity of *M. manilae* adults compared with the control group. A shorter longevity was shown in *M. manilae* adults after pupal storage at a lower temperature for a longer time, and we also recorded that the longevity of wasp adults after prepupa or late-aged pupal storage for 5 d (F = 34.21; *df* = 6, 10; *p* < 0.001) was significantly shorter than that of middle-aged pupal storage at both 4 and 10 °C. However, no significant differences were shown in adult longevity between late-aged and middle-aged pupal cold storage for 20 d (F = 76.51; *df* = 6, 6; *p* < 0.001) at both 4 and 10 °C. Moreover, the longevity of wasp adults after late-aged pupal chilling was not significantly different from that of prepupae cold storage for 5–10 d (F: 34.21–51.62; *df* = 6, 10; *p* < 0.001) at both 4 and 10 °C. To sum up, the middle-aged pupal cold storage at 10 °C for 5 d had the least effect on *M. manilae* adult longevity, with an average of 21.32 d. Moreover, the survival rates of wasp adults upon 4 or 10 °C pupal storage significantly decreased relative to that of adults raised at 25 °C (26.51 d) (Figure 3A–C).

#### 3.2.4. Determination of Biological Parameters in F2 *M. manilae*

In the final part of the survey, we focused on the biological parameters of F2 *M. manilae* upon different cold storage treatments in F1 pupae, mainly involving the emergence rate and female proportion. It was apparent that no significant differences (F: 0.06–0.07; *df* = 6, 98; *p* = 0.999) between different groups were evident in both of the two parameters (Table 6 and Table 7). The female proportion of F2 *M. manilae* upon different F1 pupal cold storage treatments was not remarkably different from the control. Whereas further analysis showed that the emergence rate of F2 *M. manilae* ranging from 84.77% to 87.84% significantly decreased (F: 2.88–2.96; *df* = 6, 87; *p* < 0.05) compared to that without pupal chilling (98.68%).

## 4. Discussion

As is known to all, parasitic wasps are one of the key factors in integrated pest management programs [40]. Therefore, it is critical to utilize local parasitoid resources and develop the most appropriate biological control program considering the various ecological circumstances in different areas of China [41]. The first question in this study sought to determine the field parasitism rate of *M. manilae*, a prevalent larval parasitoid of *S. frugiperda*, in different locations in the Hainan province of China, showing a field parasitism rate of 5.66–19.10%. Prior studies have noted that the primary hosts of *M. manilae* were *S. litura* and *S. exigua* [10,11], and no such investigations about the field parasitism of *M. manilae* on *S. frugiperda* have been conducted. In 2020, we firstly discovered that *M. manilae* successfully parasitized *S. frugiperda* in the Hainan Province of China. Our findings were very encouraging in demonstrating that the *M. manilae* acted as a potential biological control agent to control *S. frugiperda* in China, which greatly expanded the flexibility of biological control strategies using *M. manilae*.

Our next objective of the study was to determine the biological parameters of *M. manilae*. It was found that feeding on 10% sucrose water significantly prolonged the survival time of wasp adults compared to sterile water. Additionally, the longevity of *M. manilae* adults remarkably increased with the decreasing temperature from 30 to 20 °C. These results accord with the previous observations in *Tetrastichus howardi* (Olliff) [42], *Psyttalia incise* (Silvestri), *Microplitis tuberculifer* (Wesmael) [43] and *Anagrus nilaparvatae* (Pang & Wang) [44]. However, the impact of a reduced temperature on adults of *M. manilae* has not been well investigated. Considering that the host insects were optimal vectors for wasp mass-breeding [45,46], the developmental stages of both host larvae and parasitoid adults should be taken into account to achieve maximum reproductive efficiency [47,48]. It was discovered that *M. manilae* showed the highest parasitic potential on the first larvae of *S. frugiperda* and failed to parasitize the fourth instar *S. frugiperda* larvae, which was due to the elevated immune resistance to wasp parasitization in elderly host larvae and the difficulty in penetrating into the host body [49]. In accordance with the present results, the phenomenon extended to the *M. tuberculifer*-*S. exigua* and *Microplitis* sp.-*S. litura* models [50,51]. Simultaneously, we recorded the highest parasitic ability in wasp adults after 1–3 d eclosion, also supporting the previous studies of *M. tuberculife* [52] and *M. mediator* [44]. This part of the study is of great significance in clarifying the biological control potential of *M. manilae* on the control of *S. frugiperda* and provides some theoretical support for the development and breeding of parasitoids.

Cold storage is essential for maintaining a stable and adequate parasitoid population in pest management, thus contributing to its synchronized field release [18]. It has been demonstrated that the cold storage above 0 °C slowed down the metabolic rate of parasitoids and thus delayed their growth and development [17,53]. Although the obvious advantages in improving the efficiency and flexibility of large-scale release programs, the adverse effects on insect quality were worrying after cold storage [19], and a large number of studies have emphasized that the exposure of parasitoids to a chilling condition led to excessive consumption of energy reserves, thus affecting their quality and successful application in the field [20,54]. Accordingly, our next question in this study sought to determine the effects of cold storage on the biological parameters of *M. manilae* compared with the control group reared at optimal temperature conditions. Our results indicated that the emergence rate of F1 *M. manilae* after pupal cold storage was significantly lower than that of the control, showing the fitness cost in F1 caused by pupal chilling. Meanwhile, the decrease in storage temperature from 10 to 4 °C and a prolonged storage time from 5 to 10–20 d led to remarkable declines in the emergence rate of *M. manilae*. This was consistent with previous studies referring to two other species of Braconidae, *P. incisi* and *Diachasmimorpha longicaudata* (Ashmead) [16,55]. Additionally, we found that the emerged adults upon middle-aged pupal chilling showed superior performance in emergence rate compared with those of prepupae and late-aged pupal cold storage under the same storage scheme. Similarly, a study conducted on *P. incisi* showed that prepupae and late pupae were less tolerant to low temperature, showing lower emergence rates than the control group and middle-aged pupal cold treatment group [56]. Furthermore, we found that the prepupae failed to emerge after 20 d cold storage at 4 or 10 °C, which indicated its fatal effect. We proposed that parasitoids need to accumulate energy to strengthen the muscle contraction during the eclosion process [57], and low-temperature exposure for a long time led to the muscle dysfunction [58], possibly accounting for the unsuccessful eclosion of *M. manilae*. Another important finding of this study was that the longevity and survival rate of *M. manilae* adults significantly reduced with the extensions of storage time and the decrease in storage temperature. It was noteworthy that the F1 longevity and survival rate upon middle-aged pupal cold storage was significantly higher than those of prepupal and late-aged pupal treatments under the same storage conditions, which was also evidenced by previous studies [38]. Further, there were remarkable decreases in adult longevity and survival rate after pupal cold storage compared with the control group raised at optimal conditions, further demonstrating the adverse effects of cold chilling on insect physiological activity.

In addition, the reproductive system of parasitoids is extremely vulnerable to cold stress. For instance, the fertility of parasitoid wasps generally declined with the decreased storage temperature and extended storage time [26]. In our present study, the parasitism rate of *M. manilae* adults significantly decreased after pupal cold storage compared with the wasps raised at 25 °C. This result supported the idea that cold chilling led to the disorder of the insect reproductive system. It was also shown that the *M. manilae* adults after middle-aged pupal cold storage performed better than those of prepupal and late-aged pupal cold storage at both 4 and 10 °C, and these results are in good agreement with previous investigations [25,29,59,60]. In some insects, cold exposure also changed their F2 sex ratio [61]. However, no evidence of the differed female proportion was found in F2 *M. manilae* between the cold storage groups, and the control group reared at optimal temperature conditions, which indicated the effects of pupal cold storage on F2 progeny were negligible. This finding provides important cues for the mass-breeding of *M. manilae* after pupal cold storage.

Parasitoids have been widely utilized for the biological control of agricultural pests, and their mass storage is indispensable for subsequent large-scale production and release [53]. Therefore, establishing an optimum storage strategy to increase their breeding efficiency has long been the focus of studies, of which cold storage has been increasingly used [20]. However, minimizing the loss of quantity in parasitoids has become an obstacle to balancing the effects of cold storage and field application efficiency [53]. The present study aimed to clarify the effects of pupal cold storage on the biological parameters of *M. manilae*, a dominant parasitoid of *S. frugiperda* in Hainan Province of China, and provide a reliable basis for optimizing its cold storage scheme. Our results showed that middle-aged pupal storage at 10 °C for 5 d had minimal impact on the adult quality and reproductive performance in comparison with the control group reared at optimal temperature conditions. Taken together, these findings provide significant theoretical support for the commercialization of breeding and large-scale field release of *M. manilae* against noctuid pests.

## Figures and Tables

**Table 1:** Field parasitism rate of *M. manilae* in different areas of Hainan Province, China.

Collection Time	Location	Coordinate	Number of Collected *S. frugiperda*	Number of Parasitized Larvae	Parasitism Rate (%)
2020.11.10	Dongfang	108°64′ E, 18°84′ N	199	38	19.10
2020.11.03	Ledong1	108°91′ E, N18°47′ N	131	18	13.74
2020.10.23	Qiongzhong	109°90′ E, N19°13′ N	131	16	12.21
2020.11.01	Sanya	109°18′ E, N18°38′ N	238	15	6.30
2020.10.30	Ledong2	108°83′ E, N18°51′ N	53	3	5.66
2021.05.13	Danzhou	109°48′ E, N19°51′ N	235	19	8.09
2021.05.10	Sanya	109°18′ E, N18°38′ N	81	0	0.00
2020.10–2021.05	In total		1068	109	10.21

**Table 2:** Longevity of *M. manilae* adults fed different food at different temperatures.

Food	Survival Time (d)					F	*p*
	20 °C	22.5 °C	25 °C	27.5 °C	30 °C		
10% sucrose water	29.66 ± 0.98 a	28.24 ± 0.81 ab	26.51 ± 1.18 b	18.09 ± 0.55 c	12.26 ± 1.04 d	206.05	<0.001
Sterile water	6.31 ± 0.68 e	5.22 ± 0.42 ef	4.75 ± 0.37 ef	4.06 ± 0.12 fg	2.39 ± 0.44 g		

Note: Data are presented as mean ± SE (standard error) (*n* ≥ 15). Different lowercase letters indicate significant differences at the 0.05 level according to the Fisher LSD test (two-way ANOVA).

**Table 3:** Emergence rate of *M. manilae* after pupal cold storage treatments.

Storage Time (d)	Storage Temperature (°C)	Emergence Rate (%)			F	*p*
		Prepupa	Middle-Aged Pupa	Late-Aged Pupa		
5	4	65.66 ± 1.01 e	82.32 ± 0.50 c	74.13 ± 1.40 d	108.95	<0.001
	10	76.55 ± 2.11 d	91.88 ± 0.21 b	83.33 ± 0.00 c		
	25 (control)	98.68 ± 0.94 a				
10	4	26.26 ± 4.40 f	63.64 ± 0.00 cd	47.22 ± 2.78 e	138.02	<0.001
	10	58.33 ± 0.00 d	74.88 ± 1.21 b	65.66 ± 1.01 c		
	25 (control)	98.68 ± 0.94 a				
20	4	0 f	27.27 ± 0.00 c	19.39 ± 0.61 e	1027.39	<0.001
	10	0 f	36.56 ± 1.93 b	29.29 ± 2.02 c		
	25 (control)	98.68 ± 0.94 a				

Note: Data are presented as mean ± SE (*n* ≥ 15). Different lowercase letters indicate significant differences at the 0.05 level according to the Fisher LSD test (two-way ANOVA).

**Table 4:** Parasitism rate of *M. manilae* adults after pupal cold storage treatments.

Storage Time (d)	Storage Temperature (°C)	Parasitism Rate (%)			H	*p*
		Prepupa	Middle-Aged Pupa	Late-Aged Pupa		
5	4	23.08(23.08–26.92) f	42.31(38.46–42.31) d	26.92(26.92–26.92) e	83.12	<0.001
	10	50.00(46.15–53.85) c	69.23(65.38–73.08) b	53.85(50.00–57.69) c		
	25 (control)	88.46(84.62–92.31) a				
10	4	7.69(7.69–11.54) d	30.77(26.92–34.62) c	11.54(11.54–11.54) d	69.64	<0.001
	10	34.62(34.62–38.46) c	61.54(57.69–65.38) b	38.46(34.62–42.31) c		
	25 (control)	88.46(84.62–92.31) a				

Note: Data are presented as median (Q1–Q3) (*n* ≥ 15). Different lowercase letters indicate significant differences at the 0.05 level according to the Kruskal–Wallis H test (Nonparametric test).

**Table 5:** Longevity of *M. manilae* adults after different pupal cold storage treatments.

Storage Time (d)	Storage Temperature (°C)	Survival Time of Adults (d)			F	*p*
		Prepupa	Middle-Aged Pupa	Late-Aged Pupa		
5	4	8.22 ± 1.05 e	13.58 ± 0.11 d	10.54 ± 0.47 de	34.21	<0.001
	10	14.00 ± 0.08 cd	21.32 ± 0.03 b	17.87 ± 1.80 c		
	25 (control)	26.51 ± 1.18 a				
10	4	6.63 ± 0.63 e	10.88 ± 0.43 d	6.39 ± 0.72 e	51.62	<0.001
	10	12.87 ± 0.14 cd	18.78 ± 0.86 b	16.35 ± 0.65 bc		
	25 (control)	26.51 ± 1.18 a				
20	4	-	5.30 ± 0.70 c	3.88 ± 0.63 c	76.51	<0.001
	10	-	13.32 ± 0.12 b	10.63 ± 0.38 b		
	25 (control)	26.51 ± 1.18 a				

Note: Data are presented as mean ± SE (*n* ≥ 15). The same lowercase letters indicate no significant differences at the 0.05 level according to the Fisher LSD test (two-way ANOVA).

**Table 6:** Emergence rate of F2 *M. manilae* after different F1 pupal cold storage treatments.

Storage Time (d)	Storage Temperature (°C)	Emergence Rate (%)			F	*p*
		Prepupa	Middle-Aged Pupa	Late-Aged Pupa		
5	4	86.62 ± 1.41 b	87.84 ± 1.70 b	85.47 ± 1.17 b	2.96	0.011
	10	85.69 ± 1.42 b	85.51 ± 1.84 b	86.37 ± 1.89 b		
	25 (control)	98.68 ± 0.94 a				
10	4	86.17 ± 1.51 b	87.74 ± 1.57 b	86.55 ± 1.64 b	2.88	0.013
	10	86.76 ± 1.53 b	84.77 ± 1.81 b	86.63 ± 1.54 b		
	25 (control)	98.68 ± 0.94 a				

Note: Data are presented as mean ± SE (*n* ≥ 15). The same lowercase letters indicate no significant differences at the 0.05 level according to the Fisher LSD test (two-way ANOVA).

**Table 7:** Female proportion of F2 *M. manilae* after different F1 pupal cold storage treatments.

Storage Time (d)	Storage Temperature (°C)	Female Proportion (%)			F	*p*
		Prepupa	Middle-Aged Pupa	Late-Aged Pupa		
5	4	68.12 ± 3.29 a	67.21 ± 2.87 a	67.67 ± 3.26 a	0.06	0.999
	10	67.01 ± 2.97 a	68.42 ± 4.38 a	66.62 ± 3.64 a		
	25 (control)		68.82 ± 1.46 a			
10	4	67.33 ± 4.96 a	68.06 ± 2.51 a	68.93 ± 4.38 a	0.07	0.999
	10	68.59 ± 4.42 a	67.73 ± 3.88 a	66.12 ± 3.75 a		
	25 (control)		68.82 ± 1.46 a			

Note: Data are presented as mean ± SE (*n* ≥ 15). The same lowercase letters indicate no significant differences at the 0.05 level according to the Fisher LSD test (two-way ANOVA).

## Data Availability

The data presented in this study are available from the corresponding author on reasonable request.
